# Attenuation of Proinflammatory Responses by *S*-[6]-Gingerol via Inhibition of ROS/NF-Kappa B/COX2 Activation in HuH7 Cells

**DOI:** 10.1155/2013/146142

**Published:** 2013-06-16

**Authors:** Xiao-Hong Li, Kristine C. Y. McGrath, Van H. Tran, Yi-Ming Li, Colin C. Duke, Basil D. Roufogalis, Alison K. Heather

**Affiliations:** ^1^Heart Research Institute, Newtown, NSW 2042, Australia; ^2^Faculty of Pharmacy, University of Sydney, Camperdown, NSW 2006, Australia; ^3^Department of Endocrinology, Dezhou People's Hospital, Dezhou, Shandong 253014, China; ^4^School of Medical and Molecular Biosciences, University of Technology, P.O. Box 123, Ultimo, NSW 2007, Australia

## Abstract

*Introduction*. Hepatic inflammation underlies the pathogenesis of chronic diseases such as insulin resistance and type 2 diabetes mellitus. *S*-[6]-Gingerol has been shown to have anti-inflammatory properties. Important inflammatory mediators of interleukins include nuclear factor **κ**B (NF**κ**B) and cyclooxygenase 2 (COX2). We now explore the mechanism of anti-inflammatory effects of *S*-[6]-gingerol in liver cells. *Methods*. HuH7 cells were stimulated with IL1*β* to establish an *in vitro* hepatic inflammatory model. *Results*. *S*-[6]-Gingerol attenuated IL1*β*-induced inflammation and oxidative stress in HuH7 cells, as evidenced by decreasing mRNA levels of inflammatory factor IL6, IL8, and SAA1, suppression of ROS generation, and increasing mRNA levels of DHCR24. In addition, *S*-[6]-gingerol reduced IL1*β*-induced COX2 upregulation as well as NF**κ**B activity. Similar to the protective effects of *S*-[6]-gingerol, both NS-398 (a selective COX2 inhibitor) and PDTC (a selective NF**κ**B inhibitor) suppressed mRNA levels of IL6, IL8, and SAA1. Importantly, PDTC attenuated IL1*β*-induced overexpression of COX2. Of particular note, the protective effect of *S*-[6]-gingerol against the IL1*β*-induced inflammatory response was similar to that of BHT, an ROS scavenger. *Conclusions*. The findings of this study demonstrate that *S*-[6]-gingerol protects HuH7 cells against IL1*β*-induced inflammatory insults through inhibition of the ROS/NF**κ**B/COX2 pathway.

## 1. Introduction

Hepatic inflammation underlies the pathogenesis of chronic diseases such as insulin resistance, type 2 diabetes mellitus, atherosclerosis, and nonalcoholic fatty liver disease (NAFLD) [1, 2]. Liver inflammation leads to the secretion of proinflammatory cytokines and chemokines which, in turn, contribute to a feed-forward amplification of the inflammatory signal and subsequent progression of these chronic diseases [3]. Nuclear factor kappa B (NF*κ*B) is the master regulator of the hepatic inflammatory response. Under basal conditions, NF*κ*B is present in the cytoplasm of hepatocytes in a latent form. Upon exposure to proinflammatory stimuli, NF*κ*B is activated and migrates to the cell nucleus where it directs transcription of target genes [4]. These include genes encoding cytokines, chemokines, and the enzyme cyclooxygenase 2 (COX2) [5]. Cyclooxygenase (COX) is among the most important proinflammatory mediators and COX2 is responsible for persistent inflammation [6]. 

Ginger has been known for centuries as a valuable medicinal herb having anti-inflammatory properties [7, 8]. Different studies have shown that ginger extracts suppress inflammation through inhibition of the classical NF*κ*B pathway in various cell types and tissues [9, 10]. We have recently shown that *Zingiber officinale* suppressed hepatic NF*κ*B activation thereby suppressing cytokine expression from the liver [11]. Phenolic gingerols and related compounds, which are responsible for the pungent taste of ginger, have been a major focus of research related to the anti-inflammatory effects of ginger. *S*-[6]-Gingerol (1-[4′-hydroxy-3′-methoxyphenyl]-5-hydroxy-3-decanone) is the major pungent principle of ginger, with numerous pharmacological properties including anti-inflammatory and antioxidant properties [12–14]. However, the mechanisms that underlie the anti-inflammatory effects of *S*-[6]-gingerol in cytokine-stimulated hepatocytes remain largely unknown. 

In the present study, we investigated the anti-inflammatory properties of *S*-[6]-gingerol in HuH7 cells stimulated by the cytokine, IL1β. Our findings show that *S*-[6]-gingerol protects HuH7 cells against an IL1β-induced inflammatory response by inhibiting the reactive-oxygen-species- (ROS-) activated NF*κ*B/COX2 pathway.

## 2. Materials and Methods

### 2.1. Materials and Cell Culture

 [6]-*S*-Gingerol (1-[4′-hydroxy-3′-methoxyphenyl]-5-hydroxy-3-decanone) was isolated from total ginger extract as described previously [15]. IL1β was purchased from Bio-Scientific Pty. Ltd. (Gymea, NSW, Australia). N-(2-Cyclohexyloxy-4-nitrophenyl)-methane sulfonamide (NS-398), pyrrolidine dithiocarbamate (PDTC), nitroblue tetrazolium (NBT), and butylated hydroxytoluene (BHT) were purchased from Sigma-Aldrich Pty. Ltd (Castle Hill, NSW, Australia). Human hepatocyte HuH7 cells (Health Science Research Resources Bank, Osaka, Japan) were cultured in DMEM medium (Sigma-Aldrich, Castle Hill, NSW, Australia) with 10% FBS (Invitrogen, Kilsyth, Victoria, Australia) at 37°C in 5% CO_2_. 

### 2.2. CellTiter-Blue Cell Viability Assay

HuH-7 cells were seeded in 96-well plate in 100 *μ*L cell culture media and incubated for 24 hours with *S*-[6]-gingerol (50 *μ*M, 100 *μ*M, and 200 *μ*M) or DMSO (vehicle control). Following incubation, 20 *μ*L of CellTiter-Blue Reagent (Promega, Madison, USA) was added to each well with shaking for 10 sec. After incubation for another 2 hours, the plate was shaken for 10 sec and the fluorescence was recorded at 560 nm and 590 nm, respectively, according to the manufacture's protocol.

### 2.3. Transient Cell Transfections and Luciferase Measurements

HuH7 cells were transfected using Effectene (Qiagen, Hilden, Germany) with NF*κ*B-luciferase reporter vector and pRL-TK for transfection control (Promega Corporation, Madison, WI, USA), as previously described [16]. The transfected cells were preincubated with *S*-[6]-gingerol for 6 h then stimulated with IL1β for 3 h. Luciferase and Renilla activities were detected by assaying cell lysates using the Dual-Luciferase Reporter System (Promega), according to the manufacturer's protocol. 

### 2.4. RT-qPCR

Total RNA was extracted from HuH7 cells using TRI reagent (Sigma-Aldrich), and the concentration was normalized to 100 ng/*μ*L using Nanoveu (LifeScience). cDNA was generated from 100 ng of total RNA using iSCRIPT (BioRad, Regents Park, NSW, Australia). An aliquot of each cDNA sample (1 *μ*L) was amplified by real-time PCR in reaction mixtures containing primers (12 pmol each) and iQ SYBR Green Supermix (BioRad). Sequences of the primers used in the real-time PCR reaction were as follows: human IL-6 forward: 5′-CAA ATT CGG TAC ATC CTC GAC GGC, reverse: 5′-GGT TCA GGT TGT TTT CTG CCA GTG C; human IL-8 forward: 5′-CGG AAG GAA CCA TCT CAC TGT, reverse: 5′-GGT CCA CTC TCA ATC ACT CTC A; human SAA1 forward: 5′-CCA ATC ACT TCC GAC CTG CTG, reverse: 5′-GCT TTG TAT CCC TGC CCT GAG; human β2-microglobulin (B2M) forward: 5′-CAT CCA GCG TAC TCC AAA GA, reverse: 5′-GAC AAG TCT GAA TGC TCC AC. DHCR24 PCR primers were purchased from Qiagen Pty Ltd (Doncaster, VIC, Australia). Amplification was performed in a BioRad iQ5 thermocycler (BioRad) using the following protocol: 95°C for 30 sec, *T*_*m*_ of specific primer sets for 30 sec, and 72°C for 30 sec. Relative changes in mRNA levels were determined by the ΔΔCT method [17], using human B2M as the reference gene. 

### 2.5. Measurement of Intracellular ROS Generation

NBT reduction assay was used to measure intracellular superoxide anion. HuH7 cells were seeded in 12-well plates. After treatment, cells were washed with Hanks balanced salt solution (HBSS, Sigma-Aldrich) and then incubated in Krebs-Henseleit buffer (Sigma-Aldrich) containing 1 mg/mL NBT for 1 h at 37°C. Cells were then washed with HBSS and lysed with phosphate buffer (80 mM, pH 7.8) containing sodium dodecyl sulfate (SDS) and gelatin. The cell lysate was centrifuged for 5 min at 13,000 ×g and the absorbance measured at 540 nm (formazan) and 450 nm. The relative concentration of superoxide anion was calculated based on the amount of formazan formed according to the standard curve.

### 2.6. Statistical Analysis

Data are expressed as mean ± SEM. Differences between two different conditions were determined by one-way ANOVA with Bonferroni's posttest analysis used to determine significance. GraphPad PRISM Software Version 4.03 (GraphPad Software, Inc., San Diego, CA) was used for analyses. Significance was set at *P* < 0.05.

## 3. Results

### 3.1. Cell Viability

**Figure 1:**
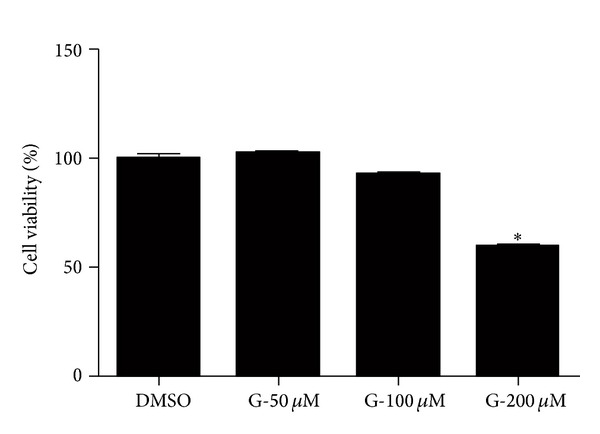
100 *μ*M *S*-[6]-gingerol is not toxic to HuH-7 cells. HuH-7 cells were exposed to *S*-[6]-gingerol (G) at different concentrations (50 *μ*M, 100 *μ*M, and 200 *μ*M) for 24 hours, and cell viability was determined using the CellTiter-Blue cell viability assay. Results are expressed as mean ± SEM of 3 independent experiments, relative to DMSO vehicle control. *P < 0.05 versus DMSO vehicle control.

To determine if *S*-[6]-gingerol affected cell viability at the concentration used in the study, cell viability assays were performed. HuH-7 cells were treated with 50 *μ*M, 100 *μ*M, or 200 *μ*M *S*-[6]-gingerol for 24 hours, and cell viability was determined using the CellTiter-Blue cell viability assay.  Figure 1 demonstrated that up to 100 *μ*M *S*-[6]-gingerol did not have a cytotoxic effect for 24 hours with a 6.88 ± 1.06% decrease in cell viability compared to DMSO vehicle control (*P* > 0.05). 

### 3.2. *S*-[6]-Gingerol Suppresses IL1β-Induced Inflammatory Cytokine Expression in HuH7 Cells

**Figure 2:**
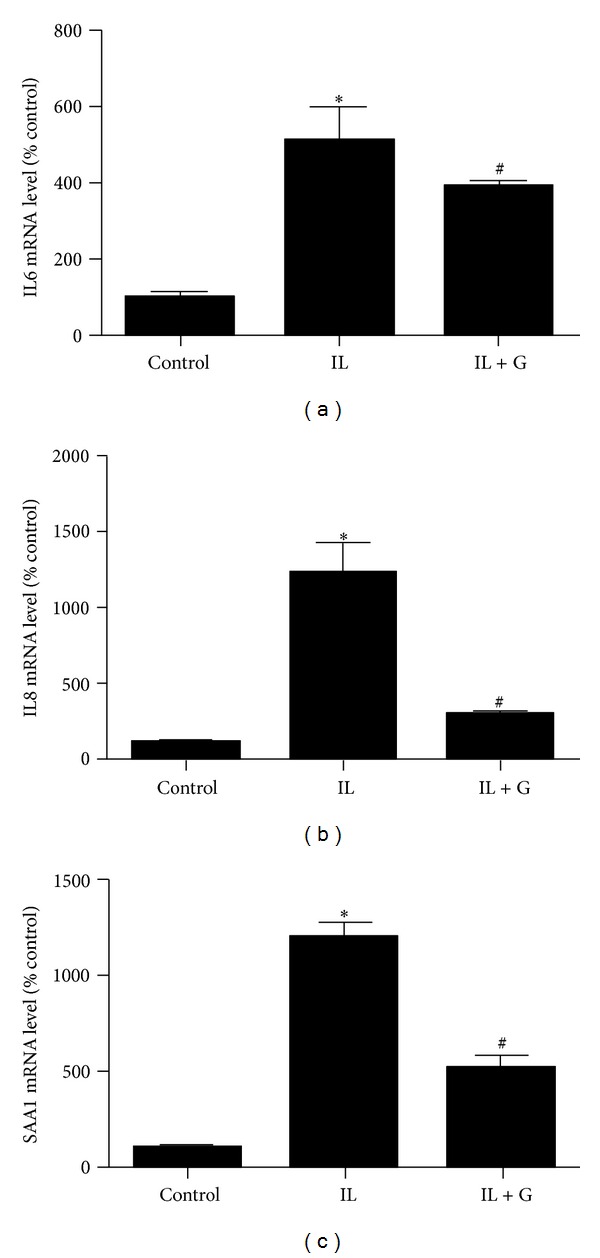
*S*-[6]-Gingerol decreases IL1β-induced inflammatory factors expression in HuH7 cell. HuH7 cells were pretreated with 100 *μ*M *S*-[6]-gingerol (G) or DMSO (vehicle control) for 6 h then stimulated with IL1β (8 ng/mL) for 3 h. The mRNA levels of IL6 (a), IL8 (b), and SAA1 (c) were measured using RT-qPCR, normalised to B2M. Results are expressed as mean ± SEM of 3 independent experiments, relative to DMSO controls. *P < 0.05 versus DMSO vehicle control; ^#^ *P* < 0.05 versus IL1β treatment.

To investigate the effect of *S*-[6]-gingerol on IL1β-induced inflammation, mRNA levels of cytokines IL6, IL8, and SAA1 were determined by real-time PCR. After exposure of HuH7 cells to 8 ng/mL IL1β for 3 h, mRNA levels of IL6 (Figure 2(a)), IL8 (Figure 2(b)), and SAA1 (Figure 2(c)) were significantly increased. Pretreatment with 100 *μ*M *S*-[6]-gingerol for 6 h before exposure to IL1β markedly suppressed IL6 (Figure 2(a)), IL8 (Figure 2(b)), and SAA1 (Figure 2(c)) mRNA levels in HuH7 cells, respectively. These results suggest that *S*-[6]-gingerol has an anti-inflammatory effect in IL1β-stimulated HuH7 cells.

### 3.3. *S*-[6]-Gingerol Inhibits IL1β-Induced Oxidative Stress in HuH7 Cells

**Figure 3:**
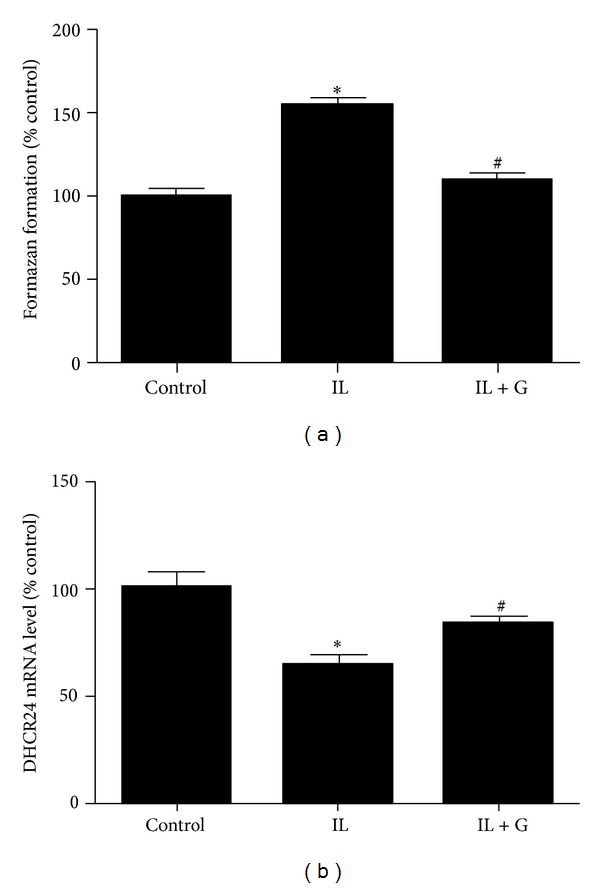
*S*-[6]-Gingerol inhibits IL1β-induced oxidative stress in HuH7 cells. HuH7 cells were pretreated with 100 *μ*M *S*-[6]-gingerol (G) or DMSO (vehicle control) for 6 h then stimulated with IL1β (8 ng/mL) for 3 h. (a) The levels of ROS generation were measured using the NBT reduction assay. The relative concentration of ROS was calculated based on the generated amount of formazan. (b) The mRNA levels of DHCR24 were measured using RT-qPCR, normalised to B2M. Results are expressed as mean ± SEM of 3 independent experiments, relative to DMSO controls. *P < 0.05 versus DMSO vehicle control; ^#^ *P* < 0.05 versus IL1β treatment.

To determine the effect of *S*-[6]-gingerol on IL1β-induced oxidative stress, intracellular superoxide and DHCR24 levels were measured. Exposure of HuH7 cells to 8 ng/mL IL1β for 3 h led to a noticeable increase in superoxide generation (Figure 3(a)). Pretreatment with 100 *μ*M *S*-[6]-gingerol for 6 h prior to IL1β exposure decreased intracellular superoxide levels in HuH7 cells. Additionally, treatment of HuH7 cells with 8 ng/mL IL1β for 3 h significantly decreased DHCR24 mRNA levels (Figure 3(b)), indicating that IL1β treatment impairs cellular defense mechanisms. Importantly, pretreatment with 100 *μ*M *S*-[6]-gingerol attenuated the inhibitory effect of IL1β on DHCR24 levels in HuH7 cell. These findings suggest that *S*-[6]-gingerol exerts an antioxidative effect on IL1β-stimulated HuH7 cells. 

### 3.4. Downregulation of Induced COX2 Contributes to the Anti-Inflammatory Effects of *S*-[6]-Gingerol in IL1β-Stimulated HuH7 Cells

**Figure 4:**
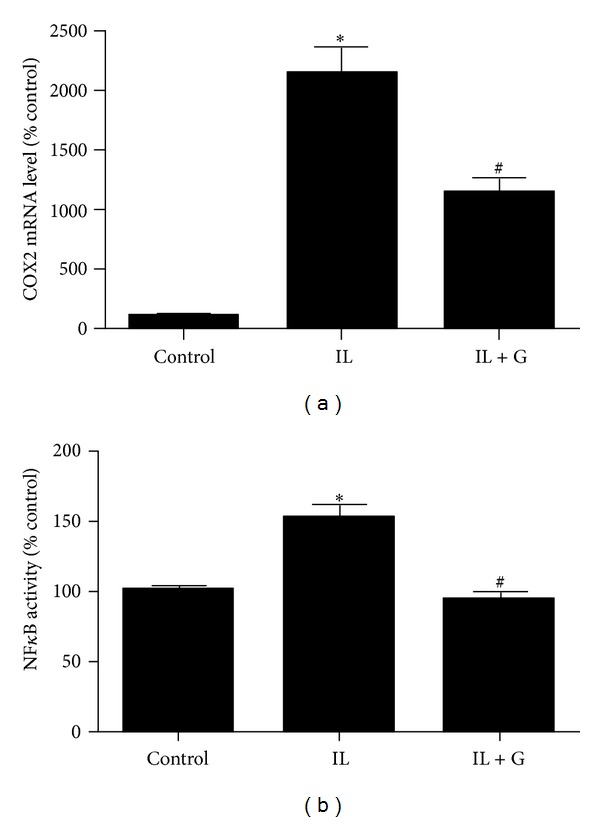
*S*-[6]-Gingerol suppresses IL1β-induced COX2 expression and NF*κ*B activity in IL1β-stimulated HuH7 cells. (a) HuH7 cells were pretreated with 100 *μ*M *S*-[6]-gingerol (G) or DMSO (vehicle control) for 6 h then stimulated with IL1β (8 ng/mL) for 3 h. The mRNA levels of COX2 were measured using RT-qPCR, normalised to B2M. (b) HuH7 cells were transfected with the NF*κ*B-luciferase reporter vector. Transfectants were preincubated for 6 h with 100 *μ*M *S*-[6]-gingerol (G) or DMSO (vehicle control) and then stimulated with IL1β for 3 h. Cells were then harvested and cell lysates assayed for luciferase activity. Results are expressed as mean ± SEM of 3 independent experiments, relative to the DMSO vehicle controls. *P < 0.05 versus DMSO vehicle control; ^#^ *P* < 0.05 versus IL1β treatment.

**Figure 5:**
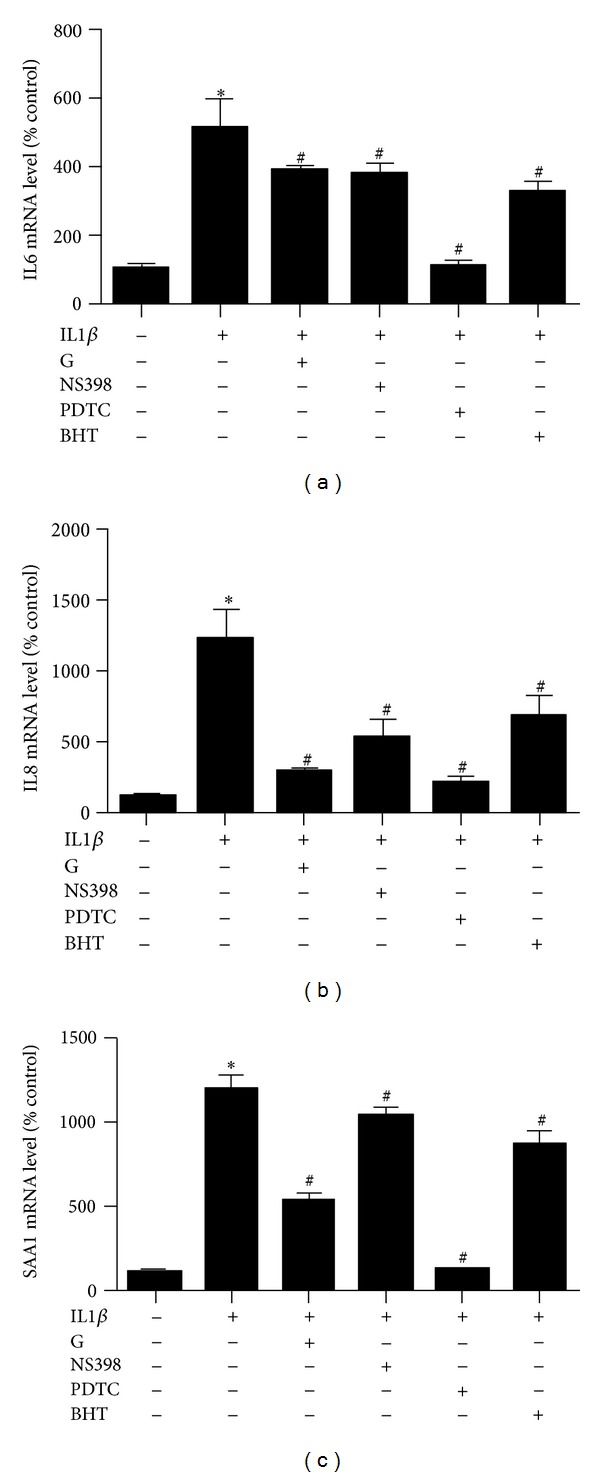
Comparison of the effects of *S*-[6]-gingerol with those of selective inhibitors on increased IL6, IL8, and SAA1 expression induced by IL1β in HuH7 cells. Before treatment with IL1β (8 ng/mL) for 3 h, HuH7 cells were pretreated with 100 *μ*M *S*-[6]-gingerol (G) for 6 h, or with 100 *μ*M NS398, 50 *μ*M PDTC, or 50 *μ*M BHT for 30 min. The mRNA levels of IL6 (a), IL8 (b), and SAA1 (c) were detected using RT-qPCR, normalized to B2M. Results are expressed as mean ± SEM of 3 independent experiments, relative to DMSO controls. *P < 0.05 versus DMSO vehicle control; ^#^ *P* < 0.05 versus IL1β treatment.

After treatment of HuH7 cells with 8 ng/mL IL1β for 3 h, expression of COX2 was significantly increased. Pretreatment with 100 *μ*M *S*-[6]-gingerol for 6 h attenuated the upregulation of COX2 mRNA levels induced by IL1β (Figure 4(a)). Furthermore, pretreatment with 100 *μ*M NS-398, a selective inhibitor of COX2, for 30 min before exposure to IL1β also inhibited mRNA levels of IL6, IL8, and SAA1 (Figure 5). These findings suggest that inhibition of IL1β-induced COX2 expression is involved in the protective effect of *S*-[6]-gingerol in HuH7 cells.

### 3.5. *S*-[6]-Gingerol Inhibits IL1β-Induced NF*κ*B Activation in HuH7 Cells

**Figure 6:**
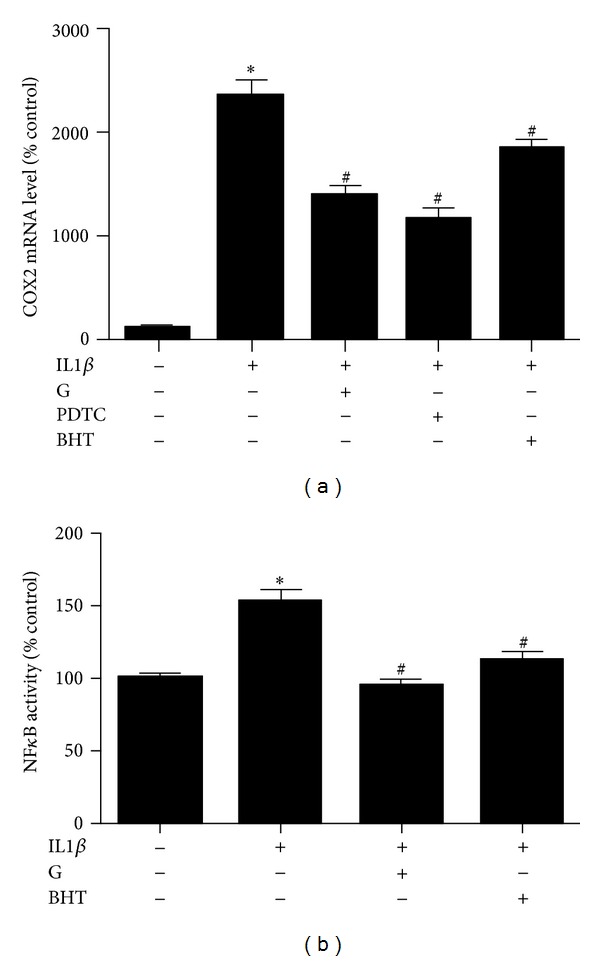
Comparison of the effects of *S*-[6]-gingerol with those of selective inhibitors on IL1β-induced COX-2 expression and NF*κ*B activity in HuH7 cells. (a) Before treatment with IL1β (8 ng/mL) for 3 h, HuH7 cells were pretreated with 100 *μ*M *S*-[6]-gingerol (G) for 6 h, or with 50 *μ*M PDTC or 50 *μ*M BHT for 30 min. The mRNA levels of COX2 were detected using RT-qPCR, normalized to B2M. (b) HuH7 cells were transfected with the NF*κ*B-luciferase reporter vector. Before treatment with IL1β (8 ng/mL) for 3 h, transfected HuH7 cells were pretreated with 100 *μ*M *S*-[6]-gingerol (G) for 6 h, or with 50 *μ*M BHT for 30 min. Cells were then harvested and cell lysates assayed for luciferase activity. Results are expressed as mean ± SEM of 3 independent experiments, relative to the DMSO vehicle controls. *P < 0.05 versus DMSO vehicle control; ^#^ *P* < 0.05 versus IL1β.

Exposure of HuH7 cells to 8 ng/mL IL1β for 3 h significantly enhanced NF*κ*B activity (Figure 4(b)). Pretreatment of HuH7 cells with *S*-[6]-gingerol for 6 h significantly inhibited IL1β-induced NF*κ*B activity. In keeping with this result, pretreatment with 50 *μ*M PDTC, a selective inhibitor of NF*κ*B, for 30 min before exposure to IL1β abrogated IL1β-induced COX2, IL6, IL8, and SAA1 expression (Figures 6(a) and 5). Together, these results suggest that the protective effects of *S*-[6]-gingerol against IL1β-induced inflammation are, at least in part, associated with inhibition of NF*κ*B activation in HuH7 cells.

### 3.6. Inhibition of Oxidative Stress Is Involved in the Protection of *S*-[6]-Gingerol against Inflammation in IL1β-Stimulated HuH7 Cells

Decreasing oxidative stress underlies anti-inflammatory effects [18]. We therefore next compared the effect of BHT, an ROS scavenger, with *S*-[6]-gingerol in their ability to suppress IL1β-induced IL6, IL8, and SAA1 expression. As expected BHT did decrease mRNA levels of IL6, IL8, and SAA1, and we found that *S*-[6]-gingerol was equally effective (Figure 5). Furthermore, it was shown that similar to *S*-[6]-gingerol, pretreatment with 50 *μ*M BHT inhibited IL1β-induced COX2 upregulation (Figure 6(a)) and NF*κ*B activity (Figure 6(b)) in HuH7 cells. The previous results indicate that suppressed ROS levels may contribute to the protective effects of *S*-[6]-gingerol against the IL1β-induced inflammatory response in HuH7 cells.

## 4. Discussion

The present study demonstrates that *S*-[6]-gingerol has the potential to protect hepatocytes against IL1β-induced inflammatory response. Moreover, for the first time, we show that *S*-[6]-gingerol achieves its potent anti-inflammatory effects through inhibition of an ROS-activated NF*κ*B/COX2 pathway. These results may explain how ginger improved insulin resistance in high-fat diet rat in our previous study [11].

COX2 has an important role in the inflammatory process. There is extensive evidence based on *in vivo* and *in vitro* models of inflammation that COX2 and COX2-induced production of prostaglandins (PGs) are involved in inflammation. Animal models of inflammation have demonstrated that an increase in COX2 mRNA and protein, as well as PG levels, parallels the inflammatory process [19]. COX2 specific inhibitors and monoclonal antibody to prostaglandin E2 (PGE2) have been shown to control inflammation [20, 21]. A study on chronic liver disease has shown that endoplasmic reticulum stress induced by hepatitis B virus X protein (HBx) enhanced COX2 expression in HBx transgenic mice and in the HuH7 cells that were transfected with HBx expression plasmid [22]. COX2 is usually absent under basal conditions, but it is inducible by various cytokines, growth factors, and mitogens [23–26]. It has been shown that the inducible COX2 is not only responsible for production of PGs in response to inflammation [24, 25], but also a potent proinflammatory mediator that promotes the production of proinflammatory cytokines including IL6 and IL1β [27]. Treatment with a selective COX2 inhibitor blocks the secretion of proinflammatory cytokines IL6, IL1β, and IL8 induced by cobalt chloride (CoCl_2_) in human skin keratinocytes [28].

Due to its proinflammatory effects, COX2 is a chief target for the treatment of inflammation. For example, the therapeutic effects of the nonsteroidal anti-inflammatory drug, aspirin, target the selective inhibition of COX2 [29]. Based on previous studies that have used IL1β-stimulated HuH7 liver cells [30–32], we too established this *in vitro* hepatocyte cell inflammatory model to investigate the anti-inflammatory mechanisms of *S*-[6]-Gingerol. The markers of inflammation that we tested were IL6, IL8, and SAA1, all known drivers or biomarkers of liver inflammation [33, 34]. Our results show that *S*-[6]-gingerol attenuated IL1β-induced COX2 expression in HuH7 cells, associated with a decrease in the expression of IL6, IL8, and SAA1. We also show that the level of inhibition achieved by *S*-[6]-gingerol for inhibition of the expression of IL6, IL8, and SAA1 was similar to that mediated by the COX2 inhibitor. Together, these data suggest that COX2 is most likely a central player in mediating the anti-inflammatory effects of *S*-[6]-gingerol. 

Hepatic inflammation can drive insulin resistance and the key mediator of the inflammatory response is NF*κ*B [3]. Activated hepatic NF*κ*B alone can drive insulin resistance as transgenic expression of the I*κ*B kinase (IKKβ) results in overt insulin resistance in mice fed a normal chow diet [3]. By contrast, when heterozygous IKKβ^+/−^ mice that express low levels of NF*κ*B are fed a high-fat diet or are crossed with obese *ob/ob* mice, they do not develop insulin resistance [35]. Moreover, NF-*κ*B inhibition by abrogation of liver IKK activity directly protects against insulin resistance in response to a high-fat diet in mice [36]. Together, these studies suggest that the liver is a primary site of inflammatory action responsible for the development of insulin resistance and that NF*κ*B is a central pathogenic factor underlying inflammation-induced insulin resistance. Our results show that *S*-[6]-gingerol inhibited IL1β-induced NF*κ*B activation in HuH7 cells. We also show that the ability of *S*-[6]-gingerol to suppress the expression of COX2 and IL8 was the same order of magnitude induced by treatment with the NF*κ*B inhibitor, while *S*-[6]-gingerol also significantly suppressed IL6 and SAA1 mRNA levels. These data suggest that NF*κ*B is involved in mediating the anti-inflammatory effects of *S*-[6]-gingerol. 

It is well recognized that inflammation is one manifestation of oxidative stress [37], and the pathways that generate the inflammatory factors, such as cytokines, are all induced by oxidative stress [37]. The present study shows that *S*-[6]-gingerol decreased superoxide production in IL1β-stimulated HuH7 cells. We also show that the ability of *S*-[6]-gingerol to inhibit IL1β-induced NF*κ*B activation and suppress the expression of COX2 and IL6, IL8, and SAA1 paralleled the results we obtained when we treated the cells with the ROS scavenger, BHT. Our results also show that *S*-[6]-gingerol increased the expression of antioxidant enzyme, DHCR24. Given that antioxidant enzymes are able to block NF*κ*B activation by various stimuli [38–44], our findings suggest that the anti-inflammatory effects of *S*-[6]-gingerol are mediated, at least in part, by suppressing oxidative stress. 

One limitation of this study is that the mechanisms of anti-inflammatory effects of *S*-[6]-gingerol demonstrated here are restricted to *in vitro* evidence. However, the results with the human hepatocyte cell line support previous findings in an animal model to demonstrate that the anti-inflammatory effect of *Zingiber officinale* (ginger) occurs through the NF*κ*B signalling pathway [11] and now define a mechanism of anti-inflammatory effects of *S*-[6]-gingerol through inhibition of cytokine IL1β-induced ROS/NF*κ*B/COX2 pathway that was not possible with the animal model. 

In conclusion, *S*-[6]-gingerol has been shown to inhibit IL6, IL8, and SAA1 expression in cytokine-stimulated HuH7 cells, via suppression of COX2 expression. We have also shown that the suppression of COX2 is achieved via blockade of the NF*κ*B signalling pathway. Finally, we have shown that *S*-[6]-gingerol blocks the NF*κ*B/COX2 pathway through suppressing the cytokine-induced oxidative stress. These results may open novel treatment options whereby *S*-[6]-gingerol could potentially protect against hepatic inflammation which underlies the pathogenesis of chronic diseases such as insulin resistance and type 2 diabetes mellitus.
